# Hyolithid-like hyoliths without helens from the early Cambrian of South China, and their implications for the evolution of hyoliths

**DOI:** 10.1186/s12862-022-02022-9

**Published:** 2022-05-17

**Authors:** Fan Liu, Christian B. Skovsted, Timothy P. Topper, Zhifei Zhang

**Affiliations:** 1 State Key Laboratory of Continental Dynamics, Shaanxi Key Laboratory of Early Life and Environments and Department of Geology, Northwest University, Xi’an, 710069 China; 2 Department of Palaeobiology, Swedish Museum of Natural History, Box 50007, 104 05 Stockholm, Sweden

**Keywords:** Hyolitha, Cambrian, Phylogeny, Evolution, The Chengjiang biota

## Abstract

**Background:**

A small hyolith, with a triangular operculum and a conical-pyramidal conch with a sharp apex, originally documented as *Ambrolinevitus ventricosus*, is revised based on new material from the Chengjiang biota. The operculum of ‘*Ambrolinevitus*’ *ventricosus* displays strong morphological similarities with the operculum of *Paramicrocornus* from the Shuijingtuo Formation (Cambrian Series 2), indicating that the species should be reassigned to *Paramicrocornus*.

**Results:**

Based on the unusual morphology of *Paramicrocornus*, we herein propose a new family Paramicrocornidae fam. nov. A cladistic analysis of Cambrian and Ordovician hyoliths clearly delineates hyolithids as a monophyletic group which evolved from the paraphyletic orthothecids in the early Cambrian and with Paramicrocornidae as its closest relative.

**Conclusions:**

The phylogenetic analysis, together with the distribution of hyoliths from the Cambrian to the Ordovician, reveals the presumptive evolution model of both the skeleton and soft-part anatomy of hyoliths. The Family Paramicrocornidae plays an intermediate role in hyolith evolution, representing the transitional stage in the evolution from orthothecids to hyolithids.

**Supplementary Information:**

The online version contains supplementary material available at 10.1186/s12862-022-02022-9.

## Background

Hyoliths are a common group of Palaeozoic marine invertebrates with a conical conch and a lid-like operculum, ranging from the early Cambrian to the Permian [1]. The group with calcareous shells, rapidly became one of the most abundant and important skeletal components of benthic faunas in the Cambrian [2–5]. Hyoliths are generally subdivided into two distinct groups, the Hyolithida and Orthothecida [3]. Orthothecids appear first in the fossil record and are usually composed of two simple skeletal components, a conical conch with a variable cross section and a flat, retractable operculum [4–9]. In contrast, hyolithids typically consist of four skeletal components, a cone-shaped conch, an externally fitting and folded operculum with distinct cardinal and conical shields, and a pair of curved spine-shaped helens [10–15]. However, some hyoliths have been described with unique combinations of characters that cannot be easily assigned to either of these two groups [16, 17]. For example, *Paramicrocornus* [18]*,* which has recently been documented in great detail from the Shuijingtuo Formation of South China (Cambrian Series 2), has drawn attention, as it represents a hyolithid-like genus with a folded operculum but apparently lacking helens, leading to the suggestion that this taxon could represent a possible sister group of hyolithids [19, 20].

Hyoliths are among the most numerous biomineralizing animals in the Chengjiang Lagerstätte and some taxa have been systematically described [8, 9, 21]. In this study, we examine new material of a small hyolith from the Chengjiang biota (Cambrian Series 2); originally described by Qian [22] as *Ambrolinevitus ventricosus* Qian, 1978*.* This species is numerically abundant in parts of Chengjiang biota [21, 23–25] but despite its abundance, some uncertainty over its taxonomic placement persists and it has been mentioned on a few occasions that the taxonomy of this taxon needs to be revised [21, 23]. In terms of general morphology, *‘Ambrolinevitus’ ventricosus* resembles typical hyolithids (triangular conch cross section, an operculum divided into cardinal and conical shields and with separate clavicles and cardinal processes). However, no evidence exists that indicates the presence of helens, which sets this taxon apart from typical hyolithids preserved in similar Lagerstätten such as the Guanshan [15], Spence Shale or Burgess Shale biotas [26]. After comparing the Chengjiang specimens with new collections of *Paramicrocornus* from the Shuijingtuo Formation across various localities in Hubei province, South China, *‘Ambrolinevitus’ ventricosus* we suggest, should be reassign to the genus *Paramicrocornus*. In addition, we erect a new hyolith family, the Paramicrocornidae, to encompass hyolithid-like hyoliths without helens. Our cladistic analysis suggests that the Paramicrocornidae includes the closest relatives of the Hyolithida which could be conveniently defined by the presence of helens [3, 12]. Our findings also provide a greater understanding of the early evolution of hyolithids, especially before the evolution of helens [20], the acquisition of which may have been related to adaptive filter feeding strategies seen in younger hyolithids [26].

## Materials and methods

The collection of all investigated specimens was approved by the Ministry of Land and Resources of China, and no particular licences are required for accessing these fossils which are deposited in the Early Life Institute, Northwest University, Xian.

### Hyoliths from the Chengjiang Biota

**Fig. 1:**
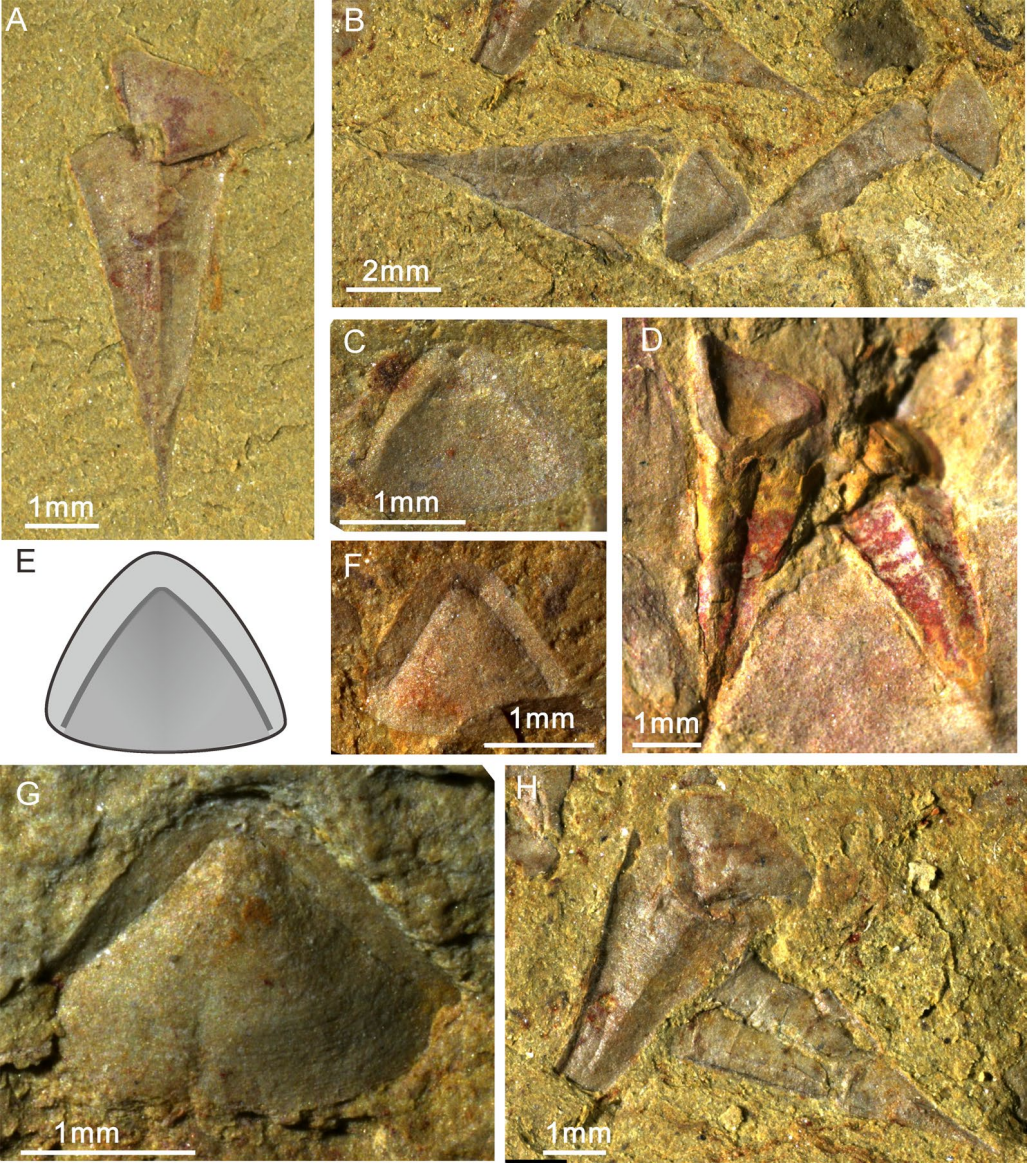
*Paramicrocornus ventricosus* from the Chengjiang Biota, Yunnan Province, South China. **A** SJZ-B16-833. General view of the morphology, note the conch with sharp apex articulated with the operculum. **B** SJZ-B18-1808. Two individuals show the complete morphology of *Paramicrocornus ventricosus* in dorsal and ventral view. **C** SJZ-B14-1719. The triangular operculum in internal view. **D** SJZ-B14-101. Articulated individuals showing the internal surface of the highly convex conical shield. **E** Sketch drawing for reconstruction of the operculum. **F** SJZ-B08-509B. The triangular operculum in external view, note the deep gap between conical and cardinal shields. **G** SJZ-B14-101. Operculum with fine, dense transverse growth lines on the external surface. **H** SJZ-B18-1808. Articulated specimens showing growth lines on the conch

**Fig. 2:**
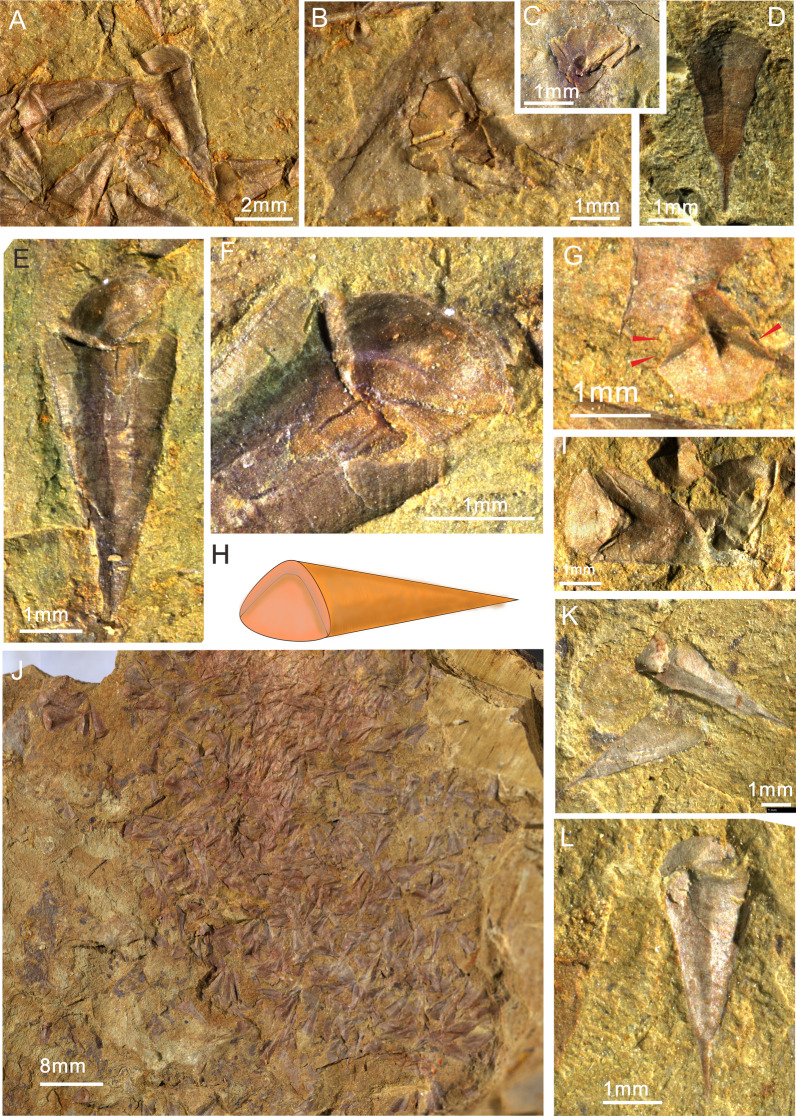
*Paramicrocornus ventricosus* preserved with some structures on the operculum from the Chengjiang Biota. **A** ELI-H-EJ186A. Complete specimens preserved in three-dimensions in dorsal view. **B** ELI-H-EJ186A. One partly buried individual covered by another animal, showing the deep sulcus between conical and cardinal shields filled with mud, and a circular pit on the summit of the conical shield. **C** ELI-H-EJ186B. Enlarged view of the counterpart of **B**. **D** SJZ-242. Conch with sharp apex and short ligula on venter. **E**, **F** ELI-H-EJ186B. **E** Articulated specimen showing the clavicles on operculum interior. **F** Close-up view of blade-like clavicles with parallel ridges representing clavicle rods. **H** Reconstruction of *P. ventricosus.* **G** SJZ-B08-509B.I Individual of *P. ventricosus* with clavicles on the internal surface of operculum marked by arrows. **I** SJZ-B08-509B. Three-dimensionally preserved specimen in dorsal view with triangular operculum. **J** SJZ-B08-509A. Aggregation of *P. ventricosus.* **K** SJZ-B04-033. Two conchs of *P. ventricosus* with sharp apex, **L** SJZ-B04-033. On articulated specimen showing the very sharp apex with a linear internal tube towards the pointed conch apex

Approximately 211 specimens of *Paramicrocornus ventricosus* (Figs. 1, 2) have been collected by the working team of the Early Life Institute of Northwest University from six different localities of the Chengjiang Lagerstätte, i.e. Chengjiang, Ercai, Erjie, Jianshan, Ma’anshan, and Sanjiezi (prefix: CJ, EC, EJ, JS, MANSH, SJZ, see Additional file 2: Table S2; for detailed locality information, Figure.1 in [27–29]), distributed on both sides of Dianchi Lake of Kunming, eastern Yunnan. The majority of samples examined here were derived from the Sanjiezi section of the Jinning area. The Chengjiang fauna is recovered from the Yu’anshan Member (*Eoredlichia* Zone) of the upper part of the Cambrian Heilinpu Formation, Cambrian Stage 3 (approximately equivalent to the Atdabanian Stage of Siberia). Most specimens in our collection were retrieved by means of splitting the mudstone along bedding planes so as to reveal casts or internal moulds of conchs, in some cases preserved with their respective opercula.

### Hyoliths from the Shuijingtuo formation

**Fig. 3:**
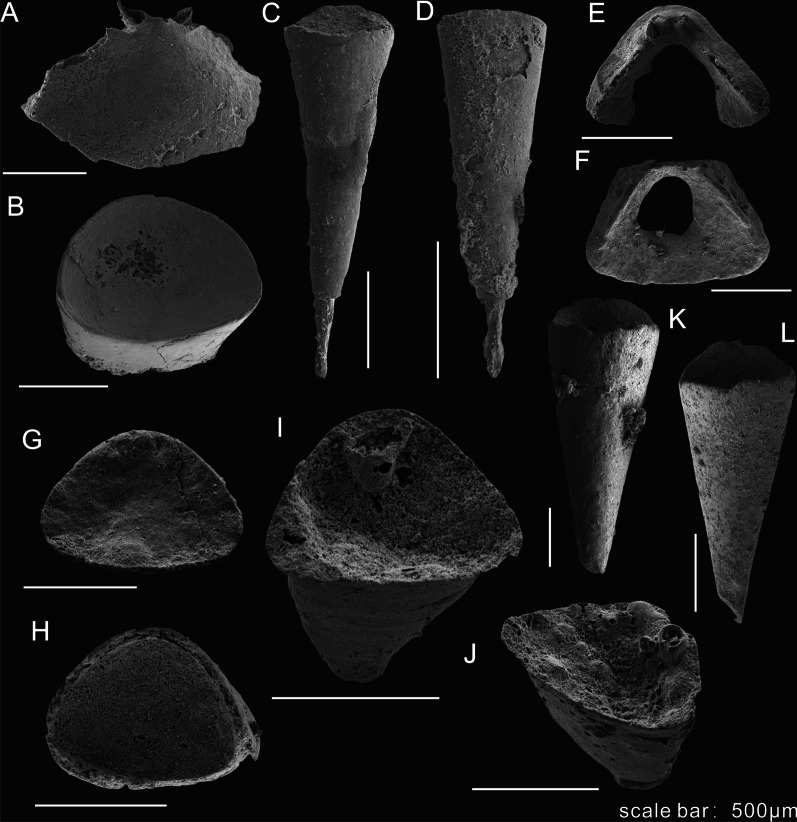
*Paramicrocornus* preserved as small shelly fossils from the Shuijingtuo Formation, early Cambrian (Series 2) in Hubei Province, China. **A**–**D** *Paramicrocornus zhenbaensis*, from the early Cambrian Shuijingtuo Formation at Xiachazhuang section, Hubei Province. **A** ELI-XCZ-SJT-5-4. Internal view of the operculum, showing the short clavicle crest and cardinal processes. **B** ELI-XCZ-SJT-5-24. Conch with triangular cross section. **C**, **D** Conchs preserved as internal moulds, showing with a linear tube towards the pointed conch apex. **C** ELI-XCZ-SJT-5-6. **D** ELI-XCZ-SJT-5-30. **E**–**L** *Paramicrocornus ventricosus* from the early Cambrian Shuijingtuo Formation at Aijiahe section, Hubei Province. **E** ELI-AJH-SJT-9-O-003. Incomplete operculum showing the triangular outline and protruding cardinal processes. **F** ELI-AJH-SJT-9-17. A partially preserved operculum in the internal view, note the short clavicle crest. **G**–**J** Conch fragments showing the equilateral triangular cross-section. **G** ELI-AJH-SJT-9-008. **H** ELI-AJH-SJT-9-011. **I** ELI-AJH-SJT-9-006. **J** ELI-AJH-SJT-9-001. **K**, **L** Partially preserved straight conchs with a short-arched ligula. **K** ELI-AJH-SJT-9-14. **L** ELI-AJH-SJT-9-15

**Fig. 4:**
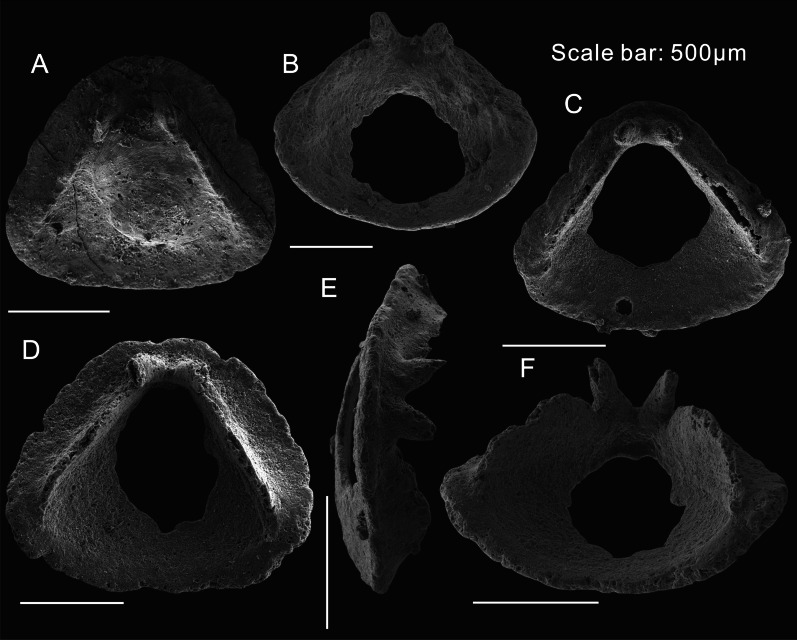
Opercula of *Paramicrocornus ventricosus* preserved as small shelly fossils from the Shuijingtuo Formation at Aijiahe section, Hubei Province*.* **A** ELI-AJH-SJT-9-10. Internal surface of a complete operculum showing triangular shape with well-preserved cardinal processes and clavicles. **B** ELI-AJH-SJT-9-O-001. Oblique lateral view of operculum, show the arcuate margin of the convex conical shield. **C**, **E** ELI-AJH-SJT-9-O-002. **C** Internal view showing a pair of strongly developed cardinal processes on a flat cardinal shield; **E** Lateral dorsal view showing the prominent internal structures. **D**, **F** ELI-AJH-SJT-9-O-004. Internal and oblique ventral views, showing the flat cardinal shield and the convex conical shield with the strong cardinal processes and clavicles

Abundant hyolith specimens of *Paramicrocornus ventricosus* and *Paramicrocornus zhenbaensis* were retrieved from small shelly fossil samples (Figs. 3, 4) collected from laminated muddy limestones interbedded with black calcareous shales from the Shuijingtuo Formation at Aijiahe and Xiachazhuang sections in the Yangtze Gorges area of western Hubei Province, South China (for locality and stratigraphic details see [19, 30, 31]). The Shuijingtuo Formation is mainly composed of black calcareous shale with concretions up to 1 m across at the base, thin-bedded organic-rich black shale in the middle and laminated bioclastic limestones in the upper part, yielding abundant fossils including brachiopods, hyoliths, trilobites, sponges, chancelloriids and some problematic organisms [19, 30]. The fossil taxa of the Shuijingtuo Formation in the Yangtze Gorges area are characterised by eodiscoid trilobites notably in the lower part and abundant acrotretid brachiopods especially in the middle–upper part [30, 32]. The trilobite biozone of *Tsunyidiscus* in the Shuijingtuo Formation is traditionally suggested to correlate with the *Eoredlichia* Zone in eastern Yunnan, which spans the strata bearing the Chengjiang biota (but see: [30, 33]). Consequently, it was suggested that the shelly taxa of the Shuijingtuo Formation are of a similar age (the Chiungchussuan Stage of the eastern Yunnan area) or slightly younger than the Chengjiang biota [30, 33].

### Methods

Specimens of *Paramicrocornus ventricosus* from the Chengjiang biota were examined and photographed using a binocular Zeiss Zoom Stereomicroscope fitted with a stereophotographic Zeiss Smart Zoom 5 camera at Northwest University. Hyoliths and other Small Shelly Fossils from the Shuijingtuo Formation at Aijiahe and Xiachazhuang sections of Hubei Province, South China were retrieved through maceration of limestone samples in acetic acid (5–10%). Hyolith specimens were handpicked from the residues, and selected specimens were coated with gold and studied using a FEI Quanta 650 scanning electron microscope (SEM) at the State Key Laboratory of Continental Dynamics, Northwest University. All specimens are housed at the Early Life Institute of Northwest University (Prefix: ELI). Cladistic parsimony analysis based on discrete morphological data (Figs. 6, 7) was performed using PAUP* version 4.0b10 [34], TNT v. 1.5. [35] (with Tree Bisection Reconnection (TBR) branch swapping), and also MrBayes 3.2.2 [36] (Additional file 1: Fig. S2) using an Mkv + Γ model with four runs each with four chains for 2,000,000 generations in the MCMC analyses and burn-in at 25%(details also in Additional file 1: Fig. S2), with an average standard deviation of split frequencies = 0.009964 and reaching convergence checked for all parameters (ESS > 200, PSRF^+^1.0) using the output of the sump command.

## Results

**Systematic palaeontology**

**Class Hyolitha Marek, 1963**

**Family Paramicrocornidae fam. nov.**

**(Figs.** **1** **,** **2** **,** **3** **,** **4** **and** **5** **)**

**Fig. 5:**
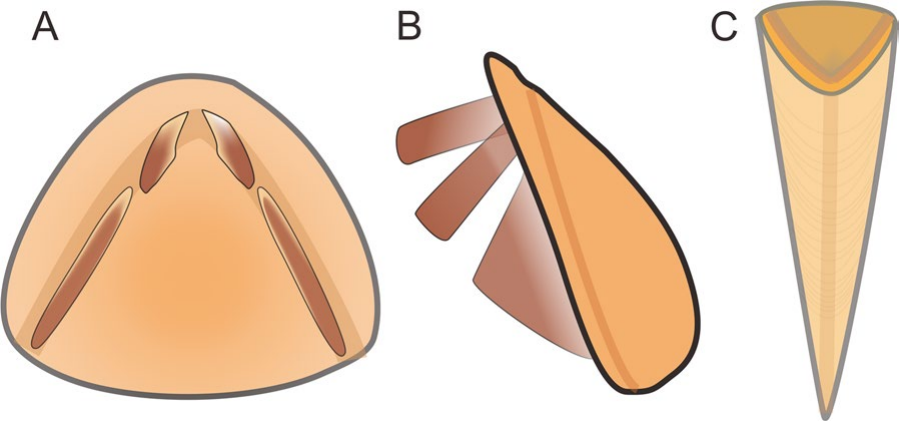
Schematic reconstruction of *Paramicrocornus ventricosus* showing the morphology of operculum and conch

**Zoobank id: 65050FC1-484C-4BD1-A762-2A825354F389**

**Type genus.** *Paramicrocornus* Qian, Xie and He, 2001 [18]; Shuijingtuo Formation; Cambrian series 2; Zhenba section, Shaanxi province, South China.

**Included genera.** Type genus and *Protomicrocornus* Pan, Skovsted, Sun & Li, 2019 [37] from Houjiashan Formation; Cambrian, Series 2, upper Stage 3 to lower Stage 4; North China.

**Diagnosis.** Hyolith with conical-pyramidal conch and externally fitting operculum without helens. Conch straight and slender with oval to triangular cross-section with short ventral ligula; gently domed dorsum; lacking lateral sinuses on the aperture. Triangular to oval operculum with distinct narrow, flat cardinal shield and highly convex conical shield; no rooflets. Cardinal processes and clavicles well developed on the internal surface of operculum along the fold formed by the cardinal and conical shields, and clavicles are formed by a series of straight rod-like units in a palisade-like arrangement. Cardinal processes and clavicles connected or separated by a narrow gap. External surface of the conch develops fine and dense transverse growth lines and the external surface of the opercula ornamented with concentric growth lines.

**Discussion.** In a recent restudy of the genus *Paramicrocornus*, Zhang et al. [19] showed that *Paramicrocornus* is compatible with hyolithids in some key features such as the distinct cardinal and conical shields of the operculum, and the possession of a slender conch with a short ligula. But *Paramicrocornus* is not a typical hyolithid, notably the taxon is lacking helens, lateral sinuses on the conch and rooflets on the operculum. It was suggested that these features indicated that *Paramicrocornus* could possibly be a member of a sister group of hyolithids rather than a hyolithid as typically defined [7, 19, 20, 38]. The closely comparable *Protomicrocornus* reported by Pan et al., [37] from the early Cambrian of North China*,* shows a similar hyolithid-like morphology without any evidence of helens, but can be distinguished from *Paramicrocornus* by the lack of a gap between the cardinal processes and clavicles on the operculum.

The genus *Paramicrocornus* was erected by Qian et al. [18] for hyoliths from the Shuijingtuo Formation, Shaanxi province, South China with a diagnostic slender conch ornamented by dense growth lines, a semi-elliptical or asymmetrically lens-shaped cross section and a venter separated from the dorsum by lateral longitudinal furrows. It was originally referred to the family Linevitidae Qian, 1989 [39], a family that consists of four genera, *Dipterygovitus, Microcornus, Trypanovitus* and *Linevitus*, with characters based on the type genus *Linevitus* Sysoev, 1958 [40]. However, the original type species of *Linevitus*, *Hyolithus obscurus* Holm, 1893 [41], from Sweden is so poorly preserved that it cannot be easily characterized. Unfortunately, the figured specimens of *H. obscurus* ([41]; pl. 5, figs. 29–30) appears to have been lost (N. Borinder, Geological Survey of Sweden, pers. com. August 2020), casting doubt on the legitimacy of the Linevitidae. The morphology of the other genera included in the family are poorly known and the family Linevitidae should be carefully revised [42]. However, at least *Microcornus* appears to have conchs with well-developed lateral sinuses, indicating the presence of helens [43], contrary to the case in *Paramicrocornus*. More recently, *Paramicrocornus* was moved to the Family Angusticornidae Sysoev, 1968 [44] by Malinky & Geyer [45] based on perceived characters such as a sharply pointed conch with keel-like lateral edges and transverse ornamentations on the shell. However, evidence from specimens of *Paramicrocornus* [19] and *Protomicrocornus* [20, 37] show no keel-like lateral edges and both genera lack helens, an obvious and distinctive difference from the other typical hyolithid genera within the Family Angusticornidae such as *Firmicornus* Sysoev, 1968; *Grantitheca* Malinky, 1988 [46]; *Nevadotheca* Malinky, 1988 [46]; *Gaka* Kruse, 1990 [47] and *Nganki* Kruse, 1990 [47]. Because of this combination of characters, it is impossible to include *Paramicrocornus* and *Protomicrocornus* in any other established hyolithid family.

**Fig. 6:**
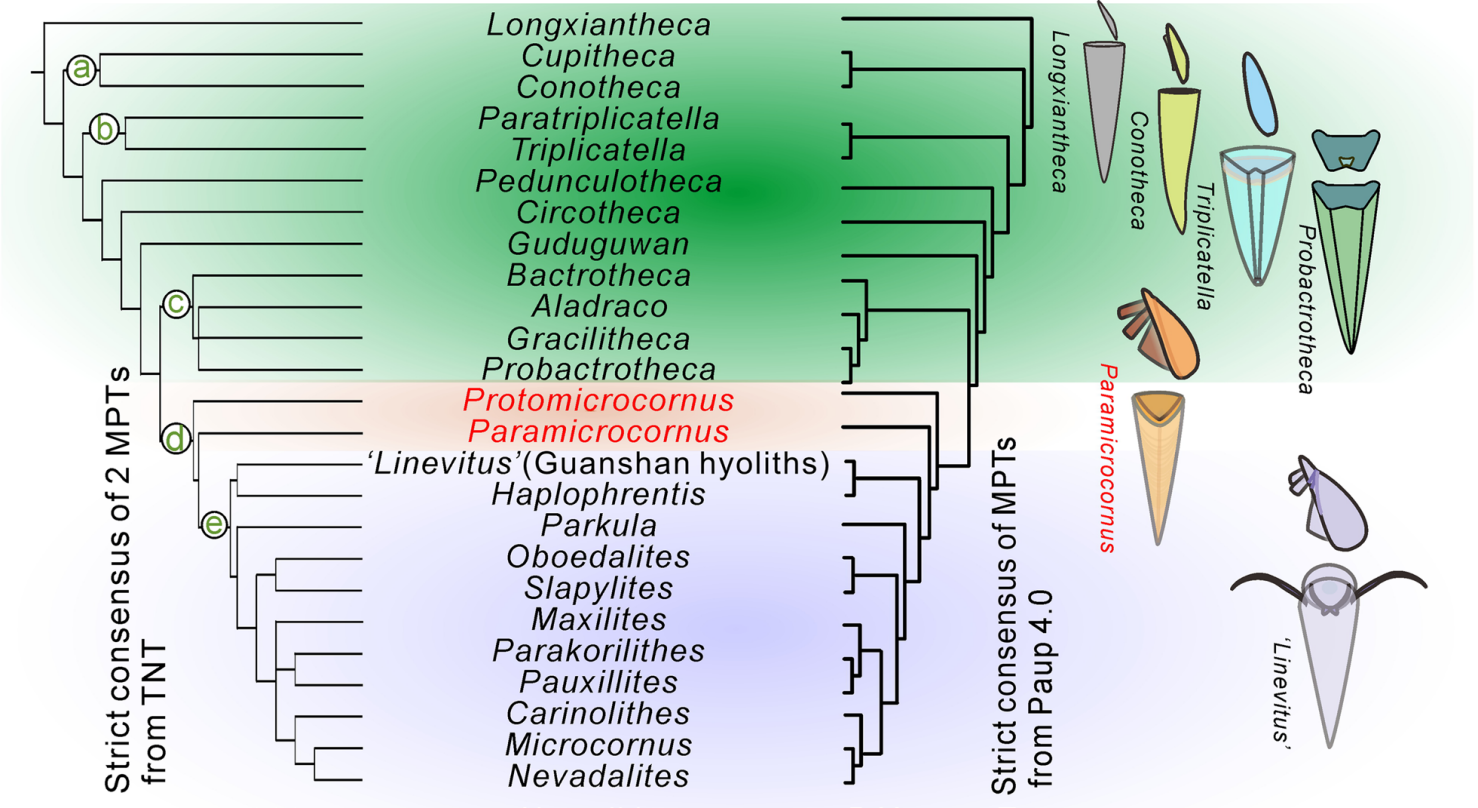
The generated tree shows the inferred phylogenetic relationships for 25 genera of hyoliths, obtained from a strict consensus tree generated from TNT v. 1.5 [35] on the left and the strict consensus generated from PAUP* version 4.0b10 [34] on the right. numbered nodes a–e (in circles) represent key synapomorphic characters. a: Slender dorsally curved conical conch; b: Presence of folds along both ventral and dorsal margin of operculum; c: Conch dorsi-ventral differentiation with angulated cross section; d: Externally fitting operculum with distinct cardinal and conical shields; e: Presence of helens or have relative features, such as lateral sinuses and rooflets. The sketchs on the right showing the evolutionary changes of the skeletal components and structures between hyolithids and orthothecids

**Fig. 7:**
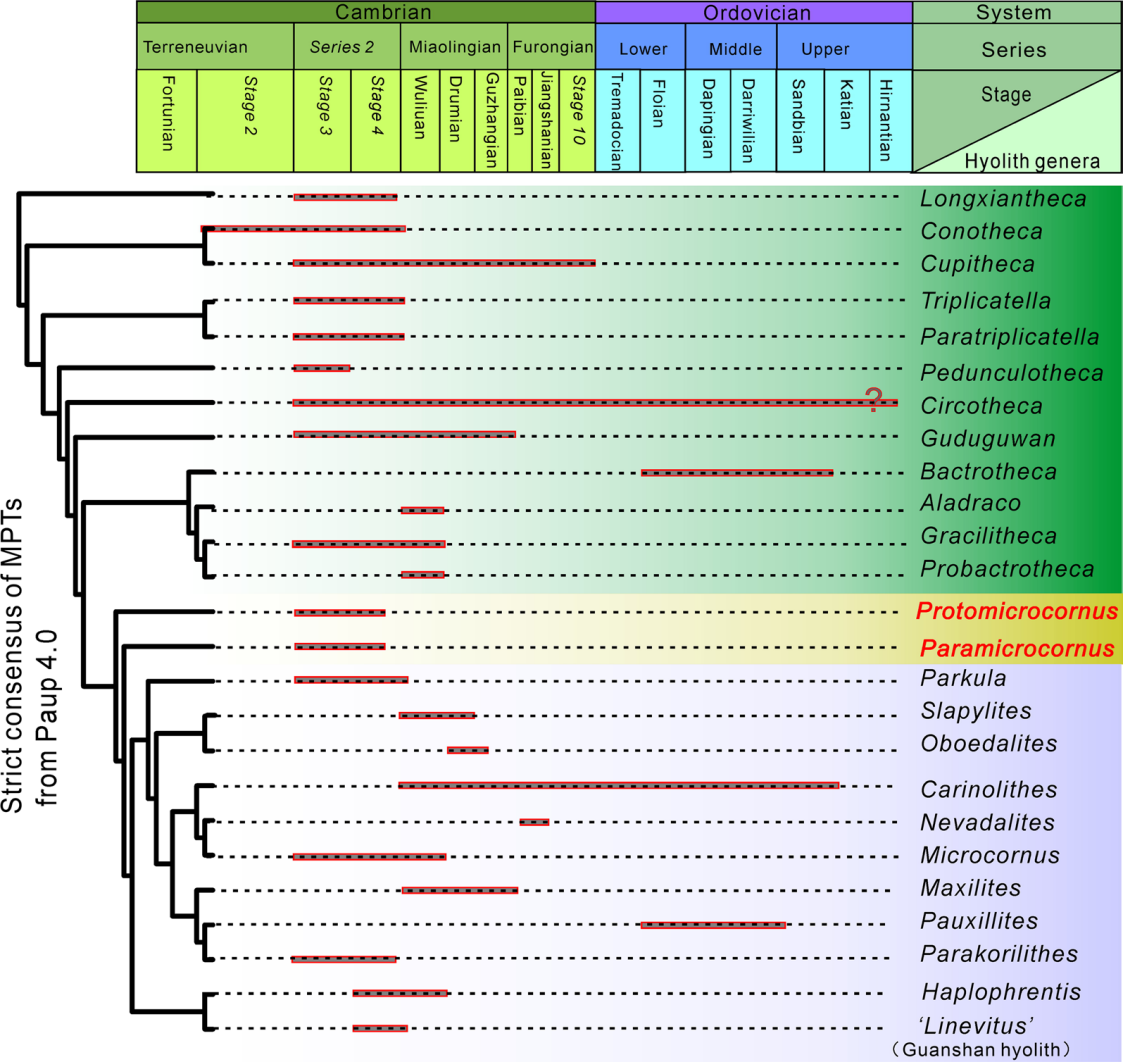
Phylogenetic position and temporal distribution of 25 genera of hyoliths during the Cambrian to Ordovician. The strict consensus of six most parsimonious tress (MPTs) generated from PAUP* version 4.0b10 [34], plotted on stratigraphy (see Additional file 1, for ages justification) (CI = 0. 490; RI = 0.695; RC = 0.340; HI = 0.510). Dark grey box with red outline shows the known distribution of hyoliths in the Cambrian and Ordovician

We here propose Paramicrocornidae fam. nov. as a new family to encompass *Paramicrocornus* and *Protomicrocornus*. The new family is differentiated from other hyolith families by a character combination of a hyolithid-like morphology (including ligula on the conch aperture and an operculum with differentiated cardinal and conical shields) but lacking helens and related structures such as lateral sinus and rooflets. The presence of helens is the key character to distinguish the hyolithids [3, 12, 20]. To clarify the phylogenetic placement of Paramicrocornidae and evaluate the rationality of the hypothetical phylogenetic position of the family [7, 19, 20, 37], we performed a preliminary phylogenetic analysis based on a dataset of 25 hyolith taxa and scored for 33 characters (Figs. 6, 7). This analysis confirms that the genera included in the new family Paramicrocornidae constitutes the closest relatives in an evolutionary lineage leading to the remaining hyolithid families (Figs. 6, 7), results and implications of this analysis are further discussed below. The characteristics uniting *Protomicrocornus* and *Paramicrocornus* (essentially the hyolithid like morphology combined with internal features on the operculum precluding the presence of helens), in our view merits the erection of a new family even though the included genera fall out in our analysis as an evolutionary grade rather than a monophyletic grouping.

**Distribution.** Early Cambrian Series 2, Stage 3–4, South and North China.

**Genus Paramicrocornus Qian, Xie and He, 2001**

**Type species.** *Paramicrocornus zhenbaensis* Qian, Xie and He, 2001 from the Shuijingtuo Formation, Zhenba section, Shaanxi province, South China.

**Revised diagnosis.** Long, straight and slender conch with initial end tapering to a sharp point. Cross section lenticular to triangular in shape with flattened or slightly convex venter and gently domed dorsum. Ventral ligula short and arcuate, dorsal aperture protruding or horizontally straight without lateral sinuses. Dorsum sometimes exhibits a median ridge. Both venter and dorsum with fine and dense transverse growth lines. Operculum triangular to oval in outline with domed, triangular conical shield, more highly convex than the flat and narrow cardinal shield. Rooflets absent and the transition between cardinal and conical shields developed as a narrow fold. Internal surface of operculum with two narrow, conjoined and protruding cardinal processes separated by a narrow gap from the blade-like clavicles extending laterally along the junction of the cardinal and conical shields. Both clavicles and cardinal processes formed by narrow sub-parallel clavicle rods. External surface of operculum ornamented with concentric growth lines.

**Remarks.** Qian et al. [18] proposed the genus *Paramicrocornus* for hyolith specimens from the Shuijingtuo Formation, Xiaoyangba section of Zhenba county, south Shaanxi Province, China, based on similarities with *Microcornus* Mambetov, 1972 [48], including a sharply pointed apex, and an ornamented conch with semi-elliptical cross section [18]. As discussed above, *Paramicrocornus* was originally referred to the family Linevitidae by Qian et al. [18] and was later included in the Family Angusticornidae by Malinky & Geyer [45]. Interestingly, the keeled lateral edges on the conch were considered as one of the diagnostic characters for the assignment of *Paramicrocornus* to the Angusticornidae [45]. These structures were described as ‘lateral longitudinal furrows’ according to the original description of *Paramicrocornus* [18]. However, combining observations of the specimens described by Zhang et al. [19] with the material recorded in the initial systematic study ([18], Plate I, figures 11–13), we conclude that the lateral transition from venter to dorsum across many specimens is smoothly rounded in outline, rather than bearing keeled lateral edges. With this in mind and considering the lack of helens, we refer the genus to the new family Paramicrocornidae, as discussed above.

The genus *Protomicrocornus* was recently reported by Pan et al. [37] from the Xinji Formation in North China and this genus appears to be closely related to *Paramicrocornus* [20, 37]. However, *Protomicrocornus* has a more rounded median ridge on the cross section of the conch and a very sharp transition between the cardinal and conical shields in the operculum, together with the lack of a gap between the cardinal processes and clavicles clearly distinguish this genus from *Paramicrocornus.*

**Stratigraphic and geographic range.** Early Cambrian Stage 3 to Stage 4; Zhenba, Shaanxi; Hubei; Yunnan; South China.

***Paramicrocornus ventricosus (Qian, 1978)***

**(Figs.** **1** **,** **2** **,** **3** **E–J,** **4** **and** **5** **).**

1978 *Ambrolinevitus ventricosus* Qian, p. 24, figs. 6.2–3 [22].

1999 *Ambrolinevitus ventricosus* Qian in Hou et al., p.86, figs. 114, 115 [24].

2000 *Ambrolinevitus ventricosus* Qian in Qian et al., p. 355–356, figs. I4–8, II6–7 [53].

2001 *Conotheca?* sp in Qian et al., p. 33, figs. II5–7 [18].

2005 *Ambrolinevitus ventricosus* Qian in Vannier & Chen, p. 12–19, figs. 8,12,13 [23].

2017 *Ambrolinevitus ventricosus* Qian in Hou et al., p. 104–105, figs. 16.2 [25].

2018 *Ambrolinevitus ventricosus* Qian in Sun et al., p. 334–338, figs. 1–2 [21].

2020 *‘Ambrolinevitus’ ventricosus* Qian in Skovsted et al., fig. 3A–B [20].

**Holotype.** ABB3-33724 from the Chiungchussu Formation, the early Cambrian, Stage 3, Yunnan Province, South China.

**Material.** In total, there are 211 specimens of *Paramicrocornus ventricosus* in our collection from the Chengjiang biota. Of these, 36 specimens are preserved as aggregates of multiple individuals, while 175 are preserved individually, sometimes articulated with operculum showing three-dimensional preservation as internal moulds. About 770 SSF specimens of *Paramicrocornus ventricosus* including opercula and conch fragments, are from the Shuijingtuo Formation in the Aijiahe section.

**Emended diagnosis.** Species of *Paramicrocornus* with slender, sharply tapering conch. Dorsum with median ridge and venter gently concave and short, arcuate ligula. Rounded triangular operculum, with distinct conical and cardinal shields. Cardinal shield with prominent cardinal processes, separated by a deep and narrow V-shaped furrow. Blade-like clavicles composed of subparallel clavicle rods along edge of conical shield reaching almost to the lateral edge of the operculum.

**Description.**

*Conch.* Small conch (mainly width 1–3.5 mm, length 4–9 mm [average length 6.36 mm, width 2.13 mm]) (see in Additional file 1: Table S2), straight with a short arch-shaped ligula on the venter (Figs. 1A, 2D, 3K, L), equilateral triangular in cross-section (Fig. 3G–J). The surface of the venter is gently concave (Figs.  1A, 2D), but the dorsum is highly inflated with a weakly developed rounded dorsal ridge (Fig. 2A, E, K). Apical angle is sharp, average about 24°. The apical region is sometimes partly filled with a central linear cavity (tube shaped) impregnated by reddish-brown stains (Fig. 2D, K, L), slowly expanding in diameter towards the aperture. The lateral margin of the conch shows a gently curved transition between the convex dorsum and the flattened venter (Fig. 3K, L, I, J). The external sculpture of densely set fine growth-lines are visible on both venter and dorsum (Figs. 1H, 2A, D, E).

*Operculum.* Triangular operculum with distinct cardinal and conical shields (Figs. 1B, D, G, H and 3. The flat cardinal shield on the posterior margin is adjacent to the conch dorsum, the arcuate margin of the convex conical shield matches the outline of the ligula on the conch venter (Fig. 1C, D). Sculpture consists of concentric growth-lines on the external surface (Fig. 1G), whereas no ornamentation was observed on the internal surface. The boundary between the conical shield and cardinal shield is apparently visible as a sulcus on the external side of the operculum (Figs. 1B, G, H; 2A, B). One specimen that is partly covered by other fossils preserves the sulcus as rod-like moulds infilling the external sulcus with sediment (Fig. 2B). A circular pit on the summit of the conical shield interior could be observed on both the part and the count-part of one specimen (Fig. 2C). In two individuals of *P. ventricosus* from the Chengjiang biota, clavicles are visibly preserved showing the characteristic blade-like structure with an ornamentation of parallel ridges (Fig. 2F, G) but no cardinal processes were observed in the material from the Chengjiang biota. The structures of the 3-dimensionally preserved opercula of *P. ventricosus* (Figs. 3E, F and 4B) from small shelly fossil assemblages show a pair of strongly developed cardinal processes on a flat cardinal shield, separated by a narrow V-shaped gap (Fig. 4A, C, D), with two blade-like clavicles aligned along the margin between the cardinal and conical shields (Fig. 4E, F). The columnar cardinal processes and clavicles are divided by an apparent deep and narrow gap (Fig. 4E, F). The structure of the clavicles is not apparent in the SSF material, and the highest part close to the gap separating them from the cardinal processes, are broken (Fig. 4B, E).

**Remarks.** *Paramicrocornus ventricosus*(Qian, 1978) was originally assigned to *Ambrolinevitus* Sysoev, 1958 [40]. The species was first reported from the lower Chiungchussu formation of Kunming, Yunnan Province [22] and the genus *Ambrolinevitus* (with the type species *Hyolithes striatellus* Holm, 1893 [41]) was originally included in the family Sulcavitidae together with the genus *Linevitus*. However, the type species upon which the genus is based with badly preserved and incomplete, rendering it almost unrecognizable and related taxa need to be reconsidered for the taxa to be redefined ([49] and p. 524 in [50]). Except for the designated type species from Sweden and the material described from Siberia [40, 51], species of *Ambrolinevitus* have only been reported from South China [22, 24, 52–54]. Following Qian [22], the diagnostic features of *Ambrolinevitus* from China are a long conch with a triangular to oval cross section, ornamented with fine growth lines, with an arched aperture and an operculum with distinct cardinal and conical shields, a generalized description that would include a multitude of hyolithid taxa. The early descriptions of *Ambrolinevitus* from China were however limited to incomplete conchs or highly compressed specimens and undoubtedly this level of preservation has hindered the identification of this taxon.

Information from the new collections from the Chengjiang biota reveal key features of both conch and operculum, confirming that it has a hyolithid-like morphology but lack evidence of helens. The morphology of specimens is reminiscent of features possessed by the small shelly fossil *Paramicrocornus* that was described from similar age strata of the Shuijingtuo Formation [19, 20]. Both set of specimens (hyoliths from the Chengjiang Biota and the Shuijingtuo Formation) share the slender, sharply tapering conch with an arcuate ligula and have a sub-triangular operculum lacking evidence of helens (or related features such as rooflets and furrow on operculum) (Figs. 1, 2, 3 and 4). All specimens from the Chengjiang biota however come from mudstones and hence have undergone high levels of compaction and the specimens show small deformations on both conch and opercula. Some specimens preserved as imprints do show the remains of some red organic stains (that have been interpreted as the remains of soft tissue in other Chengjiang hyolith taxa; see [8, 9]), while other specimens are preserved as internal moulds, with conch and operculum still articulated. Despite the mode of preservation, similarities, particularly in the morphology of opercula, displaying unique clavicles formed by a palisade arrangement of sub-parallel rods and a sharp conical-pyramidal conch with rounded lateral margins (Figs.  1, 2 and 5) suggest that all specimens can be assigned to *Paramicrocornus*.

Well preserved collections of *Paramicrocornus zhenbaensis* [18] were recently described by Zhang et al. [19] from the Shuijingtuo Formation of Shaanxi and Hubei provinces, South China. In our own SSF collections from the Shuijingtuo Formation at Aijiahe and Xiachazhuang in Hubei Province, the recovered specimens differ from *P. zhenbaensis* in shape and can instead be referred to *P. ventricosus*. Compared with *P. zhenbaensis* (Fig. 3A–D), *P. ventricosus* has a similar sharp-apex of the conch (Figs. 1A, B, 2D, L, 3K, L), and short ligula on the ventral conch aperture (Figs. 1A, 2D, L, 3K, L), as well as similar blade-like clavicles on the operculum. However, the operculum of *P. ventricosus* is triangular (Figs. 3E, F, 4 and 5) rather than oval in shape as in the type species and additionally, *P*. *ventricosus* has a visible median ridge on the dorsum and gently concave venter of the conch (Figs. 1A, 2D, L, 3K, L). The operculum of *P. zhenbaensis* is high and convex (Fig. 3A; [19], Fig. 3]), with a domed shape which is also distinct compared with the flatter and more triangular opercula of *P. ventricosus* (Figs. 3E, F, 4). Although only observable in the acid isolated SSF material, the gap separating the cardinal processes as viewed from the anterior is narrow and V-shaped in *P. ventricosus* (Fig. 4B, E, F) compared to the U-shaped gap in *P. zhenbaensis* (Fig. 3A; [19], Fig. 3]).

**Stratigraphic range and distribution.** The Yu’anshan Member of the upper part of Heilinpu Formation, early Cambrian, Stage 3, Yunnan Province, South China; The Shuijingtuo Formation, Cambrian, Series 2, Hubei Province, South China.

### Phylogenetic analysis

The establishment of Paramicrocornidae and the wealth of new data recently reported on hyolith taxonomy and morphology [7–9, 13, 14, 18–21, 26, 55, 56] necessitates an overhaul of hyolith interrelationships and for this purpose we performed a phylogenetic analysis based on Cambrian and Ordovician hyolith taxa (Figs. 6, 7, Additional file 1: Fig. S1; Additional file 3: Table S3), including both orthothecids and hyolithids as conventionally defined, as well as problematic forms, such as *Paramicrocornus*.

#### Taxa analysed

To determine the affinities of different groups of hyoliths, we selected 25 hyolith genera from the Cambrian and Ordovician for which detailed morphological information is available from published studies (Figs. 6, 7, Additional files 1, 3: Table S3). Most of the selected taxa are well-known typical hyolithids or orthothecids, preserving morphologies of both conch and opercula, which eases character coding. Eleven typical hyolithids consisting of *Haplophrentis* Babcock & Robison, 1988 [57]*, Slapylites* Marek, 1980 [58]*, Nevadalites* Marek, 1976 [59]*, Microcornus* Mambetov, 1972 [48]*, Parkula* Bengtson in Bengtson et al., 1990 [43]*, ‘Linevitus’ *(Guanshan hyoliths) [15]*, Pauxillites* Marek, 1966 [4]*, Parakorilithes* He & Pei in He et al., 1984 [60]*, Carinolithes* Sysoev, 1958 [40]*, Oboedalites* Marek, 1981 [61], *Maxilites* Marek, 1972 [62] as well as eleven typical orthothecids including *Triplicatella* Conway Morris in Bengtson et al., 1990 [43]*, Paratriplicatella* Pan, Skovsted, Sun & Li, 2019 [37]*, Conotheca* Missarzhevsky, 1969 [63]*, Cupitheca* Duan in Xing et al., 1984 [64]*, Guduguwan* Kruse, 1990 [47]*, Gracilitheca* Sysoev, 1968 [44]*, Circotheca* Sysoev, 1958 [40]*, Bactrotheca* Novák, 1891 [65]*, Probactrotheca* Valent in Valent et al., 2012 [66]*, Longxiantheca* Li in Li et al., 2020 [7]*, Pedunculotheca* Sun in Sun et al., [55] were selected*.* Two genera combining key morphological characters typical of the Hyolithida and Orthothecida; *Protomicrocornus* and *Paramicrocornus* were also added to the matrix in addition to the genus *Aladraco* which was recently described by Geyer [67] as another taxon combining hyolithid and orthothecid morphological characters.

#### Character selection and definition

In general, both the complex internal structures of the operculum [7, 37, 42] and characters of the conch, including the ligula, aperture types, lateral edges and cross section are considered important for hyolith taxonomy [37, 68]. In total 33 phylogenetic characters (Additional file 1: Table S1, Additional file 3: Table S3) pertinent to the fundamental morphology of hyoliths as outlined in previous studies were identified. These consist of 30 characters of the mineralized skeleton (conch, operculum and helens) including internal structures, and three characters relating to the soft anatomy. Each taxon was coded based on descriptions and figures of hyoliths from the literatures (see in Additional file 1, references).

#### Phylogenetic analyses

Cladistic parsimony analysis (Figs. 6, 7) was performed using PAUP* version 4.0b10 [34] and TNT v. 1.5. [35]. A Bayesian analysis using MrBayes v.3.2.2 [36] was also performed (Additional file 1: Fig. S2). Most polymorphic characters are coded as discrete numbers with different specific means as ‘0–6’ (see in Additional file 1: Table S1, Additional file 3: Table S3). Genera that have more than one character are coded with a combined number. The inapplicable characters are coded "–", and some features are unknown or unavailable in the limited fossil recorded and are consequently coded as ‘?’. All characters were treated unordered and equally weighted. Considering the uncertain phylogenetic position of hyoliths, *Longxiantheca* was selected to root the tree, which is an orthothecid with a circular conch cross section and an operculum lacking internal processes, similar to the reported morphology of the oldest known hyolith taxa [7]. The TNT analysis was undertaken using Traditional Search options, with 1000 random stepwise addition replicates followed by Tree Bisection Reconnection (TBR) branch swapping and the determined default concavity constant. The strict consensus tree is shown here including important synapomorphies (Fig. 6). Tree construction using PAUP was calculated using the heuristic search based on parsimony, followed by Tree Bisection Reconnection (TBR) branch swapping followed on 1000 additional sequence replicates. Figure 7 shows the strict consensus tree from PAUP.

#### Results of analyses

The trees generated using parsimony (the results were generated in TNT, and replicated in PAUP) and bayesian analyses are slightly different concerning the placement of some genera close to the base of the tree and within the Hyolithida but the main pattern is the same across all trees (Fig. 6, Additional file 1: Fig. S2). Notably, the Hyolithida form a monophyletic grouping evolving from a paraphyletic Orthothecida. The family Paramicrocornidae (including *Paramicrocornus* and *Protomicrocornus*) in both of trees constitutes the closest relatives and form a nested evolutionary lineage towards the Hyolithida (Figs. 6, 7).

The parsimony analyses (Figs. 6, 7) identified a basal group of hyoliths with a roughly circular cross section and slightly curved conch (*Longxiantheca, Conotheca* and *Cupitheca*) although their exact relationships differ between the PAUP and TNT analyses. The Triplicatellidae (*Triplicatella* and *Paratriplicatella*) is placed between *Pedunculotheca* and a clade consisting of *Conotheca* and *Cupitheca*. The rest of the orthothecid taxa (*Circotheca, Guduguwan, Gracilitheca, Bactrotheca, Probactrotheca*) falls in a nested lineage leading towards the Hyolithida. In our analysis, the enigmatic *Aladraco* groups with *Gracilitheca* and *Probactrotheca* in a separate clade within the paraphyletic Orthothecida (Figs. 6, 7).

The taxa traditionally referred to the Hyolithida (*Haplophrentis, Slapylites, Nevadalites, Microcornus, Parkula, ‘Linevitus’* (Guanshan hyoliths)*, Pauxillites, Parakorilithes, Carinolithes, Oboedalites, Maxilites*) represent a monophyletic group in all analyses. However, the internal relationships between the hyolithid taxa are not strongly supported and slightly differ between the analyses (Fig. 6).

## Discussion

The evolution of the Hyolitha has long been controversial. However, as the oldest known hyoliths are orthothecid-like taxa, it is widely hypothesized that hyolithids evolved from a paraphyletic Orthothecida [20]. Despite this general consensus, a phylogenetic analysis of the Hyolitha has been rarely undertaken, and typically only a few genera of hyoliths are included [55]. Over the last few years, a range of early Cambrian taxa have been discovered expressing a unique combination of characters that have challenged the established dichotomy of the Orders Orthothecida and Hyolithida [17, 19, 67, 69–71]. How exactly these unusual taxa, such as *Paramicrocornus* and *Aladraco* fit into the evolutionary history of the Hyolitha has yet to be resolved.

Our cladistic analysis of Cambrian and Ordovician hyoliths support the above hypothesis that the Hyolithida is a monophyletic group that evolved during the early Cambrian from orthothecid ancestors (Fig. 6, 7, Additional file 1: Fig. S2). The oldest orthothecids, as exemplified by *Longxiantheca mira* have frequently been considered as representing the ancestral state of hyoliths, possessing a conch with a circular cross section and a ‘simple’, round and smooth operculum, lacking in internal morphological structures [7] (Fig. 6). During the early Cambrian a series of hyolithid-like characters were acquired by orthothecids that would lead to the evolution of the Hyolithida (Fig. 6). These characters include the development of a sub-triangular cross section and dorso-ventral differentiation, and the development of internal structures of the operculum (Synapomorphies for some clades are described in the Fig. 6 with numbered character nodes (in circles)). From our analyses it appears that these characters were obtained in a step-wise fashion, as early orthothecid taxa such as *Cupitheca, Conotheca, Circotheca* and *Pedunculotheca* still possessed the ancestral tubular conch (without any distinct differentiation of venter and dorsum), yet had evolved prominent internal structures on the internal surface of the operculum (Fig. 6). Slightly younger and more derived Cambrian orthothecid taxa (Fig. 7) such as *Probactrotheca* and *Bactrotheca* moved away from possessing a tubular conch and instead developed conchs with distinct venter and dorsum (triangular to trapezoid in cross section with angulated), together with possessing an operculum with internal features, such as cardinal processes (Fig. 6, 7).

Not all orthothecid taxa included in our analyses however conform to this stepwise acquisition of hyolith-like characters. *Aladraco, Gracilitheca* and *Probactrotheca* form a distinct clade in both analyses as derived orthothecid taxa (Figs. 6, 7). While, all three taxa have developed a conch with a trapezoidal or triangular cross section, they all display relatively ‘simple’ opercula. In fact, *Aladraco* has never been found associated with an operculum and consequently this taxon has been interpreted as not possessing this characteristic feature [67]. The lack of an operculum prompted Geyer [67] to suggest that *Aladraco* represents a hitherto unrecognized animal clade derived from the hyolithids [67]. The consistent association of *Aladraco* with *Gracilitheca* and *Probactrotheca* within the paraphyletic Orthothecida in our analyses provides no evidence to support Geyer’s [67] claim. However, further research into whether the absence of an operculum in *Aladraco* is a true or taphonomic signal is necessary to clarify the phylogenetic position of this genus.

*Protomicrocornus* and *Paramicrocornus* are two of the taxa that display a unique combination of characters that has previously led to uncertainties over their higher-level taxonomy (Figs. 6, 7). For reasons stated above, we erected the Paramicrocornidae to accommodate both taxa that according to our analyses represents an intermediate grade between the orthothecids and the hyolithids (Figs. 6, 7). Our analyses indicate that the presence of the complex structures on the operculum or conch such as lateral sinuses, furrow/rooflets etc. (characters a–c), are a significant feature that separates hyolithids from orthothecids (Fig. 6). The Paramicrocornidae are united with hyolithids by characters (characters d) of the operculum (differentiation of cardinal and conical shields) (Figs. 4, 6) and the presence of a ligula on the conch aperture (Fig. 3C, K, L). Although the Paramicrocornidae have several features reminiscent of hyolithids, they are missing one key morphological feature that is here considered to represent a synapomorphy that characterizes the entire hyolithid clade and that is the development of helens (Fig. 6).

The origin of helens was discussed in detail by Skovsted et al. [20], who proposed four evolutionary stages in the development of helens. First, the retractable operculum of the ancestral orthothecid is replaced by an externally fitting operculum. This innovation was followed by the development of radial clavicle-like structures on the internal surface of the operculum and the evolution of ligula and the folded operculum. Finally, the clavicle rods detached and helens were formed. This proposed progression in the development of helens is mirrored in our analyses (the four stages exemplified by *Conotheca–Paratriplicatella–Paramicrocornus–Haplophrentis*).

The interrelationships of the hyolithids are however difficult to ascertain from our analyses as parsimony analyses show poor support within the group (Additional file 1: Fig. S1B, also seen in the Bayesian analysis Additional file 1: Fig. S2). This low level of support is most likely due to a combination of hyolithid taxa showing only subtle morphological differences and our lack of knowledge regarding the soft-part anatomy of the majority of taxa. Many genera of hyolithids in our dataset also have an uncertain higher-level taxonomy and are probably in need of taxonomic revision.

## Conclusions

With detailed revisions and new discoveries of significant hyolith taxa, the genera-level cladistic analysis herein has provided a more refined view of hyolith interrelationships, particularly in the evolutionary lineage leading to the Hyolithida. However, the analyses do not clearly resolve the earliest evolution of the group or the interrelationships of the younger orthothecids or hyolithids. As such this can only be regarded as a first step in a more complete investigation of hyolith evolution. In the future, a more extensive phylogenetic analysis is warranted, including data from more examples among the oldest known hyolith taxa and a larger sample of younger hyoliths, both hyolithids and orthothecids. Better resolution of the hyolith interrelationships would also hinge on a better understanding of the skeletal structures, particularly of the operculum. It is for example not clear how the marginal ring-like structures present in many early orthothecid opercula relate to the more extensive internal projections (cardinal processes and clavicles) identified in later hyoliths. The nature of the clavicles represents another problem as the interrelationships of the different types of clavicles (monclaviculate, biclaviculate, platyclaviculate etc.) which differentiates many hyolithid taxa, and their relationship to the palisade-like clavicles and clavicle rods of the Paramicrocornidae also remains to be explored. Finally, the identification of suitable outgroups to root the tree are required to resolve the phylogenetic position of hyoliths in the animal kingdom.

## Supplementary Information

**Additional file 1.** List of selected characters and coding comments on hyoliths in cladistic analysis.** Figure S1**. The trees generated from TNT v. 1.5.

**Additional file 2: Table S2.** Examined specimens of *Paramicrocornus ventricosus* from the Cambrian Chengjiang biota, South China.

**Additional file 3: Table S3.** The character state matrix of 25 hyolith taxa with the distribution in the Cambrian and Ordovician.

## Data Availability

All relevant data are available from the authors. All the specimens dealt with in this paper are deposited in the Early Life Institute and Department of Geology (http://geology.nwu.edu.cn/). Correspondence and requests for materials should be addressed to ZZF (elizf@nwu.edu.cn).
