# Supplementary material for: Hyolithid-like hyoliths without helens from the early Cambrian of South China, and their implications for the evolution of hyoliths
Source: BMC Ecol Evol. 2022 May 17;22:64. doi: 10.1186/s12862-022-02022-9 (PMC9116025; doi:10.1186/s12862-022-02022-9)
Supplement: Supplementary file 1 — Additional file 1. List of selected characters and coding comments on hyoliths in cladistic analysis. Figure S1. The trees generated from TNT v. 1.5. [file 12862_2022_2022_MOESM1_ESM.docx]

**Supplementary Material of**

***Hyolithid-like hyoliths without helens from the early Cambrian of South China, and their implications for the evolution of hyoliths***

Fan. Liu^1,2^, Christian B. Skovsted^1,2^, Timothy P. Topper^1,2^, and Zhifei, Zhang^1^*

^1^State Key Laboratory of Continental Dynamics, Shaanxi Key Laboratory of Early Life and Environments and Department of Geology, Northwest University, Xi'an 710069, China

^2^Department of Palaeobiology, Swedish Museum of Natural History, Box 50007, SE-104 05 Stockholm, Sweden

*correspondence should be addressed to Z F.Z [elizf@nwu.edu.cn or zhangelle@126.com]

**Characters selected:**

Conch:

1. Brephic shell: Morphology;

0: sharp tubular-liner shaped
1: Sub-sphaerical to Spherical(bulbous)
2: Fusiform

Character 1 refers to the different shapes of the apical parts of the conch [1,2]. Dzik [1] reported two types of bulbous apical parts, including sub-sphaerical and fusiform. Skovsted et al. [3], Sun et al. [4] described the larval shell of hyolith *Cupitheca* as a swollen apex with a terminal spine ([4], fig.1H, 2E), which we assigned it to the fusiform shaped*.* Some genera of hyolith show the sharp liner-tubular shaped apex of internal mould. The best example from *Aladraco schloppensis*, which described as ‘the apical portion formed by axial chamber’ ([5], fig. 3). *Paramicrocornus ventricosus* also preserved as the sharp apex showing a long liner shaped on the proximal ends of conch (Fig.2D, L). The apical structures of *Pedunculotheca diania* were illustrated as ‘pedicles’[6], which was re-descibed by Liu et al. [7] showing like the linear tube of the internal mould ([7], fig.5G, 6B).

1. Larval shell separated by nick or furrow;

0: Not extended; embryonic shell contiguous with adult shell
1: Larval shell separated from adult shell by prominent nick or furrow.

Some hyolith genera shows a clear separation of conch and larval shell in the form of a prominent nick or furrow. In other taxa the larval shell is continuous with the shell or is only delineated by the commencement of co-marginal growth [1,2].

1. Shell ultrastructure;

0 parallel fibres;

1 lamello-fibrillae;

2 lamellar(Bidirectional aragonite folia);

3 lamellar(Unidirectional aragonite folia);

4 Crossed foliated lamellar;

5 tubular pore;

6 fibrous bundles

There are 3 types shell ultrastructure in the operculum and conch of hyoliths [8], including fibrous bundles, lamello-fibrillar, parallel fibres (not sure), three types of lamellar microstructures [9] that consist of Bidirectional aragonite folia microstructure, Unidirectional aragonite folia and crossed foliated lamellar microstructure (see in new taxon described here). Kouchinsky[10] reported that the shells of hyoliths preserved a system of tubules in the channels between the bundles of the inner and outer layer, called a tubular pore [11].

1. Surface ornament -Transverse growth lines:

0 absent

1 present

1. Surface ornament -Strength of transverse growth lines:

1weak

2 strong

1. Surface ornament -Longitudinal growth lines:

0 absent

1 present

Some genera of hyoliths are ornamented with various types of growth lines, such as transverse and longitudinal lines.

For example:

Slapylites：in Valent et al. [12] p.500 ‘Lateral edges roundly sharp. External surface of the dorsal side ornamented with fine transverse anastomosing and often discontinuous fine ribs, giving the surface a reticule-like appearance. Ventral side bears only fine growth-lines’

*Pauxillites*: in Valent & Corbacho [13] p.52 ‘Dorsal sculpture consisting of fine longitudinal costae; ventral side marked with growth lines.’

Parakorilithes: in Pan et al. [14] p.374 ‘The surface ornamentation consists of prominent growth lines and faint longitudinal striations’.

Transverse \ well-defined growth lines show differences with fine ribs on the depth of the growth lines preserved on the conch. Transverse \ well-defined growth lines are weakly and tight preserved, but fine ribs show more prominent and strongly preserved.

1. Septa;

0 absent;

1 present

Septa is a structure usually on the apex of the conch which divides conchs into several chambers. Some genera of hyoliths continue to secrete septa in the apex of the adult conch, like *Haplophrentis* [16]; *‘Linevitus’malongenis* [17]; *Cupitheca* [3,4,9], *Triplicatella* showing the horseshoe-shaped apical portion [7], *Longxiantheca* [8] and *Pedunculotheca diania*[6]. In some genera of hyoliths the presence of septa is uncertain due to limitations of preservation, for example in *Paratriplicatella* [14].

1. the curvature of the conch

0: Straight- Flat venter

1: Curved - Dorsally

2: Curved - Ventrally

The apex of hyolith conch preserved as SSF in the lateral view shows curved ventrally or dorsally, some genus show the conch straight or planar in the lateral profile from opening of conch (or aperture) to apex. Some genus of hyolith preserved from Konservat-Lagerstätte which were flat after the high compression and could not sure whether the apex state curved or not.

1. cross section;

0 oval-round;

1 triangular-sub triangular;

2 cap-shaped- subtrapezoidal

The conchs of hyoliths show a wide variation of cross-sectional shapes (typically circular, rectangular or sub triangular).

1. lateral ridges;

0 absent;

1 present (weak);

2 present (strong/ sharp)

In lateral view, there are lateral ridges between the ventral and dorsal conch in some hyolith genera. Some hyolith genera on the other hand with round cross section will not possess lateral ridges on the conch.

1. ligula on the conical shell;

0 absent;

1 present (short);

2 present (long)；

1. lateral sinuses on the conch

0 absent;

1 present

Lateral sinuses are vital for the stability of the helens that extrude from the interior, at the dorso-ventral transition of the conch. Lateral sinuses of the conch are typically a perfect fit for the helens, allowing the operculum to close with the helens still protruding from the interior.

1. the dorsal margin of the conch;

0 sinous margins in lateral views (cyrtoconic /curved conch dorsally concave);

1 straight margins in lateral views, but dorsal side is regularly arched;

2 straight margins in lateral views with an apically directed indentation or prominent ridge on the dorsum

Valent et al. [18] reported *Maxilites* ‘Dorsum highly inflated; venter almost flat’

Valent et al. [12] described Slapylitidae as “cyrtoconic (curved, tapering conical conch) conch dorsally concave, dorsal side is regularly arched without any keel, lateral edges rounded or sharp”.

1. Dorsum of conch: Medial ridge;

0: absent
1: present(high/strong)

2: present(weak)

The dorsum of the conch for some hyolith genera will have keels or structures called medial ridges.

1. Dorsum of conch: furrow

0: absent

1: present (one)

2: present(two)

1. lateral and dorsal views of aperture

1 orthogonal;

2 oxygonal;

3 amblygonal;

Marek [19] distinguished several types of apertures, including orthogonal, oxygonal, amblygonal. See in Malinky and Berg-madsen [20], p.32 ‘Anorthogonal aperture is one in which the dorsal rim intersects the longitudinal axis of the conch at a right angle and is therefore straight or level. In an oxygonal aperture, the dorsal rim has a central protrusion toward the anterior end of the conch, whereas in an amblygonal aperture the dorsal rim curves to form an indentation oriented toward the apical end of the conch.’

1. the venter margin of conch;

0 venter upward vaulted showing as ventral side concave;

1 flat or plain venter

2 venter downward vaulted showing as the ventral side convex

Some genera have a flat venter which has been used to support a benthic life habit as it provided a stable surface to rest on the seafloor. Venters of other hyolith genera show as protruding outward or receding medially inward of the conch ([20], fig.3). *Probactrotheca* was described as ‘ventral side concave’([21], p.244). *Gracilitheca* is also reported with a very slightly concave venter conch ([22], p.214). Some genus of Orthothecids have a sub-circular to circular cross section and they always have a convex ventral side, such as *Circotheca* and *Conotheca*. Most hyolithids show a flat venter. *Microcornus* are reported as the ventral side weakly convex in diagnosis ([15], p.555). The venter margin of different hyolith genera could be distinguished through the cross section of conch see in the TEXT-FIG. 3 in [20].

Operculum：

1. operculum;

0 externally fitting operculum;

1 retractable operculum；

Orthothecids are normally with a more or less flat, retractable operculum without helens. On the contrary, hyolithids are considered as having an external operculum.

1. fold on the margin of operculum

0 absent;

1 present（dorsum）；

2 present（ventral）

Opercula in some genera of hyoliths bear posteriorly diverging (dorsal) folds; a single wide but very weak anterior (ventral) fold.

1. Surface ornament on operculum;

0 smooth;

1 well-defined concentric lines;

2 radial

1. Cardinal teeth

0: Absent

1: Present

Marek, 1963 described and defined the cardinal teeth of hyolith as radially arranged teeth, adorn the cardinal margin of the operculum of certain hyolithids.

Maxilites: Dorsal teeth figured in Martí Mus and Bergström [23] and Valent et al., [18].

Pauxillites: Present in [22] and [24].

Parkula: Pan et al., [14] reported *Parkula bounites* in p. 372 “Numerous, closely set teeth-like folds are often present along the dorsal margin of the cardinal shield (Fig. 11A, A1)”

Carinolithes: Valent et al., [25] described *Carinolithes bohemicus* in P.103 as “Distinct cardinal teeth are well developed in the cardinal area.”

Slapylites: Valent et al., [12] figure 2b.

1. Operculum divided into cardinal shield and conical shield;

0 No (flat operculum without structure);

1Yes

The operculum of hyolithids is usually divided into distinct cardinal and conical shields by a prominent fold, furrow or rooflets.

1. Height of cardinal shield;

1 low (narrow);

2 high

1. Placement of the operculum larval shell;

0: centre;

1: off centre

1. Rooflets;

0 absent;

1 present

The fold between the cardinal and conical shields of the operculum, are called furrow or rooflets, which are utilised for housing the protruding helens.

1. helens;

0 absent;

1 present

Helens are the third skeletal element of the typical hyolithid. They are an oar-like element that projects from the rooflets/furrow between the conch and operculum, and does not occur in orthothecids.

1. cardinal processes;

0 absent;

1 present

1. gap between cardinal process and clavicles;

0 absent;

1 present

1. clavicles:

0 absent;

1 present

1. Type of clavicles;

1 Monoclavicle;

2 Triclavicle- platyclaviculate;

3 “palisade-like”

Soft-part anatomy:

The soft-parts of hyoliths are generally only discernible in Konservat-Lagerstätte. The following three characters are based on some reports from the Cambrian [6, 7,16,17, 26-30], we recognize that due to preservational factors that these characters cannot be observed in taxa liberated from carbonates using acid digestion techniques.

1. pharynx;

0: extending beyond dorsal margin of operculum;

1: restricted to length of the operculum.

1. tentacles;

1 present (separate);

2 present (tuft-like arrangement)

1. Gut type;

1 U-shaped gut;

2 anal tube straight and loosely folded gut；

3 anal tube straight and gut straight with local zigzag folds

Table S1. Character state matrix for TNT, MrBayes and PAUP analysis of 25 hyolith taxa. Characters and character states are listed above.

Haplophrentis10?1101?111110011001101??11111111

Slapylites?0?1210?012?000(1,3)100(1,2)11110?1111???

Nevadalites ???120??011??10?10011011??1112???

Microcornus(0,1)1(2,4)1100?11112103(1,2)00?1?1?11?1?????
Parkula?041200?011?120(1,3)10011111111111???

Linevitus???0-01001111003100?1011?11111011

Pauxillites20?111??1121?00?1001?1?1??1112???

Parakorilithes20(3,4)(0,1)11020111(0,1)00220011021111112???
Carinolithes???121??0121212320011110111?12???

Oboedalites00?121??0021?00?10021011111111???

Protomicrocornus??4????02010100210211010001013???

Paramicrocornus ?0111000(0,1)010(1,2)003(0,1)001101?001113???
Aladraco00?0-100210?210?1????????0???????

Maxilites???121?00?11100?100111111?1112???

Triplicatella ???110112010(0,2)01-11(1,2)100-0000-0-022
Paratriplicatella????????2010(0,1)00211(1,2)100-0000011???
Conotheca11(1,2,5)(0,1)10110000000-210100-0001-0-??(1,2,3)

Cupitheca21(1,3,5)0-0110000000-210(1,2)00-1001-0-???

Guduguwan00?120000000100?110??0??001-0-??3

Gracilitheca ???0-1??12???10?01???????????????

Circotheca00(4,5,6)120100000100-210100-0001-0-??3
Bactrotheca11?1110?200020??110000-0001-0-???

Probactrotheca???111002200221-012100-?001-00???

Longxiantheca??60-01(0,1)0000(0,1)00-210200-0000-0-?2?
Pedunculotheca01?1201?1100100-210100-0000011???


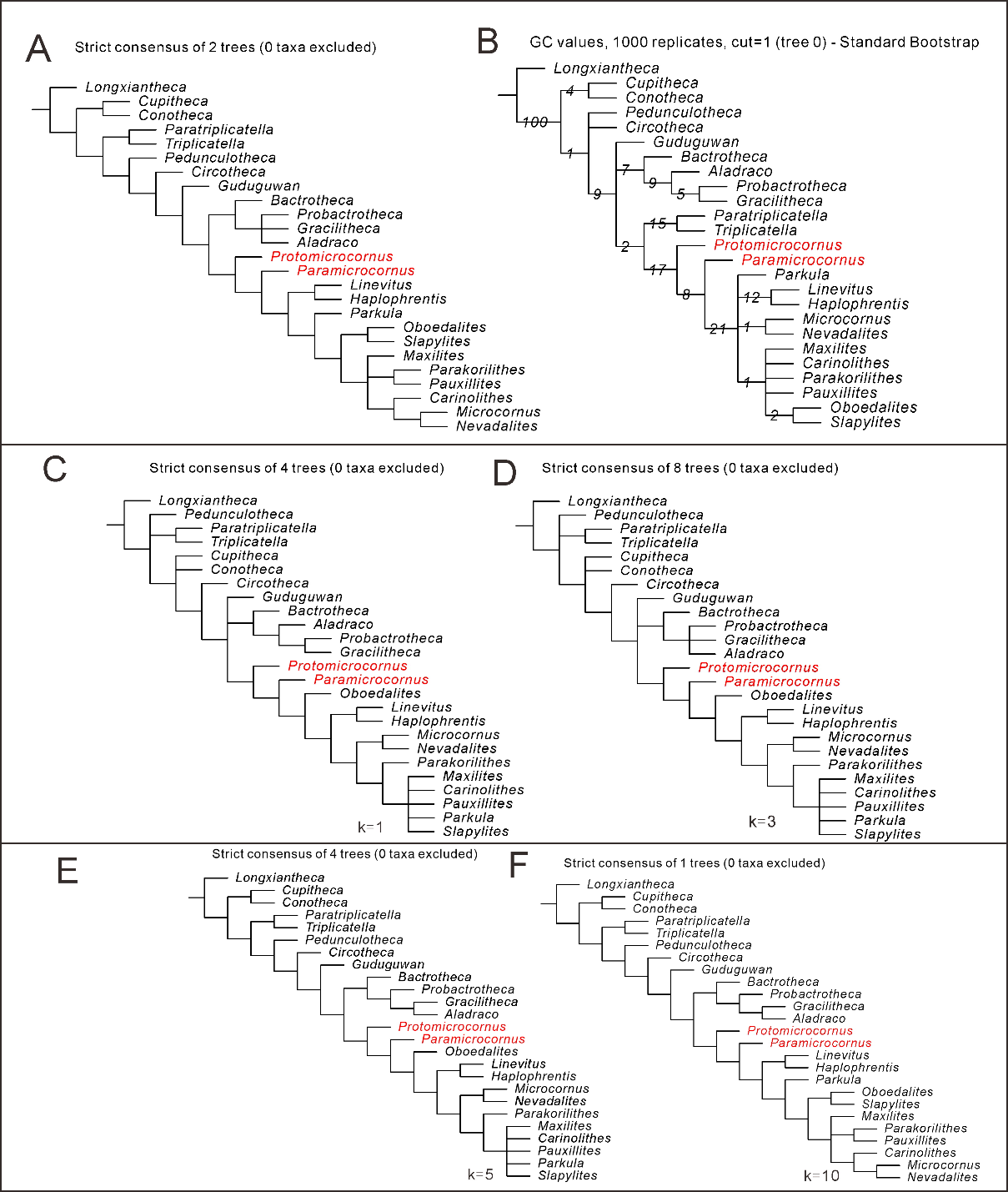


Figure S1.A, Tree search using new technology (TNT), including Ratchet and Drifting with default settings. B, The analysis of Standard Bootstrap from TNT. C-F, Strict Consensus of Tree Search analyses (TNT), using implied weighting for k= (1, 3, 5, 10).


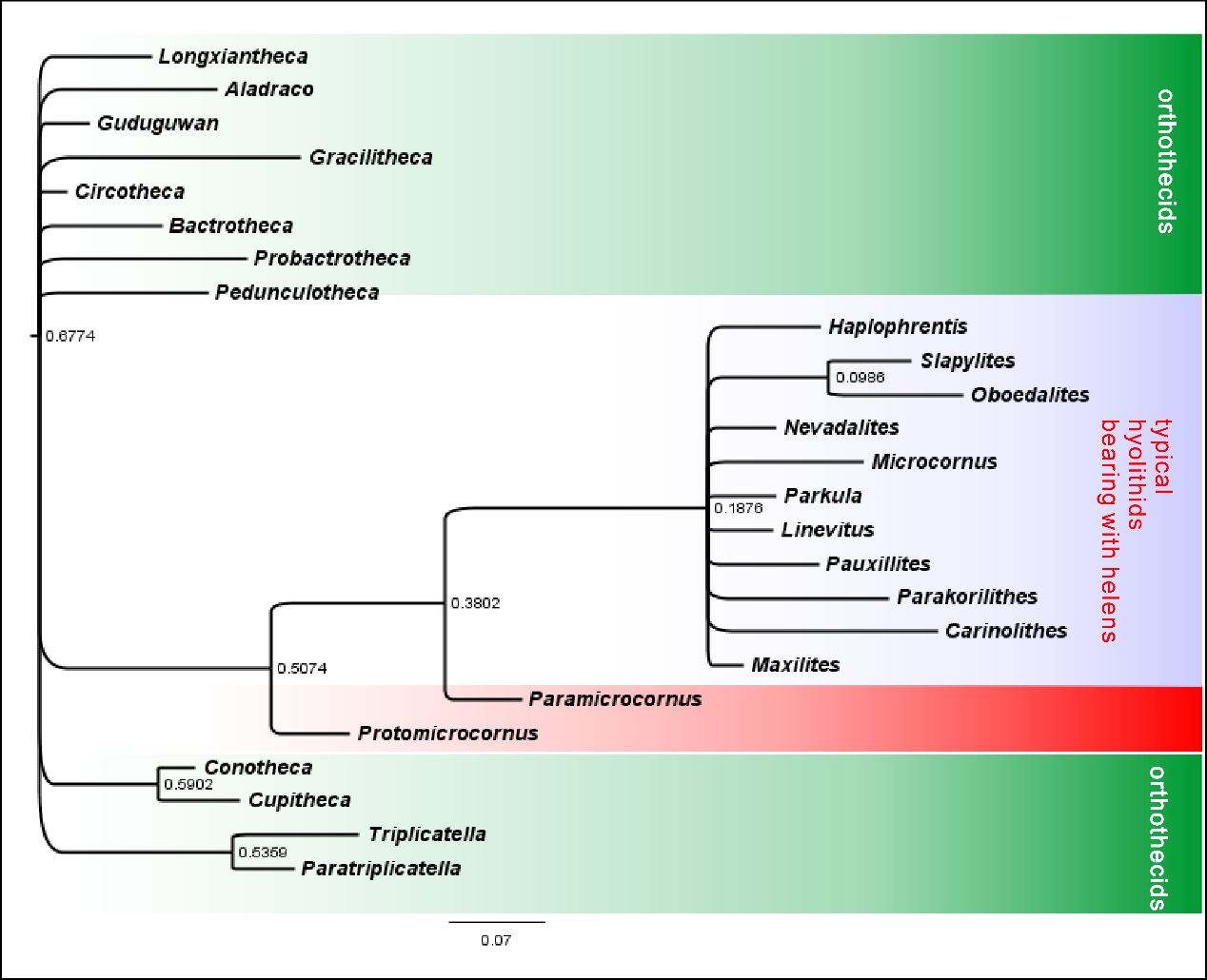


Figure S2. Maximum clade compatibility tree through Bayesian 3.2.2 analysis [31] using an Mkv+Γ model([32]) with four runs each with four chains, and not a backbone used. Numbers next to branches are posterior probabilities. 2000,000 generations were requested in the MCMC analyses. (Average standard deviation of split frequencies = 0.009964; with convergence checked for all parameters (average ESS >200, PSRF^+^1.0) using the output of the sump command).

**References**

- 1. Dzik J. Larval development of hyolithids. Lethaia 1978; 11(4): 293-299.
  2. Dzik J. Ontogeny of *Bactrotheca* and related hyoliths. Geologiska Föreningen i Stockholm Förhandlingar 1980; 102(3): 223-233.
  3. Skovsted C B, Pan B, Topper T P, et al. The operculum and mode of life of the lower Cambrian hyolith *Cupitheca* from South Australia and North China. Palaeogeography, Palaeoclimatology, Palaeoecology 2016; 443: 123-130.
  4. Sun H, Yin Z, Li G, et al. Periodic shell decollation as an ecology‐driven strategy in the early Cambrian Cupitheca. Palaeontology 2020; 63(3): 431-442.
  5. Geyer G. A new enigmatic hyolith from the Cambrian of West Gondwana and its bearing on the systematics of hyoliths. Papers in Palaeontology 2018; 4(1): 85-100.
  6. Sun HJ, Smith MR, Zeng H, et al. Hyoliths with pedicles illuminate the origin of the brachiopod body plan. Proceedings of the Royal Society B: Biological Sciences 2018; 285(1887): 20181780.
  7. Liu F, Skovsted, CB, Topper, TP, et al. Are hyoliths Palaeozoic lophophorates?. National Science Review 2020; 7(2): 453-469.
  8. Li LY, Skovsted CB, Yun H, et al. New insight into the soft anatomy and shell microstructures of early Cambrian orthothecids (Hyolitha). Proceedings of the Royal Society B 2020; 287(1933): 20201467.
  9. Li LY, Zhang XL, Skovsted C B, et al. Homologous shell microstructures in Cambrian hyoliths and molluscs. Palaeontology 2019; 62(4): 515-532.
  10. Kouchinsky A V. Skeletal microstructures of hyoliths from the Early Cambrian of Siberia. Alcheringa 2000; 24(2): 65-81.
  11. Feng WM. Preliminary research on tubular pore system of Early Cambrian Meishucunian hyolith conch. Acta Palaeontologica Sinica 2003; 42(4): 585-589.
  12. Valent M, Fatka O, Marek L. *Slapylitidae*: a new family of hyolithids (Cambrian–? Devonian; Baltica, Laurentia, Gondwana). PalZ 2017; 91(4): 497-505.
  13. Valent M, Corbacho J. *Pauxillites thaddei* a new Lower Ordovician hyolith from Morocco. Acta Mus. Nat. Pragae, Ser. B-Hist. Nat 2015;71(1-2): 51-54.
  14. Pan B, Skovsted CB, Sun HJ, et al. Biostratigraphical and palaeogeographical implications of Early Cambrian hyoliths from the North China Platform. Alcheringa: An Australasian Journal of Palaeontology 2019; 43(3): 351-380.
  15. Malinky J M, Skovsted C B. Hyoliths and small shelly fossils from the Lower Cambrian of North-East Greenland. Acta Palaeontologica Polonica 2004; 49(4).
  16. Babcock LE, Robison RA. Taxonomy and paleobiology of some Middle Cambrian Scenella (Cnidaria) and hyolithids (Mollusca) from western North America. The University of Kansas Paleontological Contributions 1988; 1–22.
  17. Liu F, Skovsted CB, Topper TP, et al. Soft part preservation in hyolithids from the lower Cambrian (Stage 4) Guanshan Biota of South China and its implications. Palaeogeography, Palaeoclimatology, Palaeoecology 2021; 562: 110079.
  18. Valent M, Fatka O, Szabad M. The oldest hyolith fauna of the Jince Formation (mid-Cambrian, Barrandian area, Czech Republic). Neues Jahrbuch für Geologie und Paläontologie-Abhandlungen 2018; 281-291.
  19. Marek L. The class Hyolitha in the Caradoc of Bohemia. Sborník Geologických Věd, Paleontologie 1967; 9: 51-112.
  20. Malinky JM, Berg‐Madsen V. A revision of Holm's Early and early Mid Cambrian hyoliths of Sweden. Palaeontology 1999; 42(1): 25-65.
  21. Valent M, Fatka O, Szabad M, et al. Two new orthothecids from the Cambrian of the Barrandian area (Hyolitha, Skryje-Týřovice Basin, Czech Republic). Bulletin of Geosciences 2012; 87(2): 241-248.
  22. Valent M, Corbacho J, Martínez D. Hyolith localities of Zagora region (Morocco), Upper Fezouata Formation (Lower Ordovician). Batalleria 2013; 19: 20-23.
  23. Marti Mus, Monica, and J. A. N. Bergström. The morphology of hyolithids and its functional implications. Palaeontology 2005; 48(6): 1139-1167.
  24. Marek, L. New hyolithid genera from the Ordovician of Bohemia. Časopis národního muzea, 1966; 135: 89-92.
  25. Valent M, Fatka O, Szabad M, et al. Carinolithidae fam. Nov., *Carinolithes bohemicus* sp. Nov. and *Slehoferites slehoferi* gen. et sp. Nov.–new hyolithid taxa from the Bohemian middle Cambrian (Skryje-Týřovice Basin, Czech Republic). Palaeobiodiversity and Palaeoenvironments 2011, 91(2): 101-109.
  26. Kruse P D. Hyolith guts in the Cambrian of northern Australia‐turning hyolithomorphs upside down. Lethaia 1996; 29(3): 213-217.
  27. Devaere L, Clausen S, Álvaro J J, et al. Terreneuvian orthothecid (Hyolitha) digestive tracts from northern Montagne Noire, France; taphonomic, ontogenetic and phylogenetic implications. PLoS One 2014; 9(2): e88583.
  28. Berg-Madsen V, Valent M, Ebbestad J O R. An orthothecid hyolith with a digestive tract from the early Cambrian of Bornholm, Denmark. GFF 2018; 140(1): 25-37.
  29. Moysiuk J, Smith MR, Caron JB. Hyoliths are Palaeozoic lophophorates. Nature 2017; 541(7637): 394-397.
  30. Liu F, Skovsted CB, Topper TP, et al. Revision of *Triplicatella* (Orthothecida, Hyolitha) with preserved digestive tracts from the early Cambrian Chengjiang Lagerstätte, South China. Historical Biology 2020; 1-15.
  31. Ronquist F, Teslenko M, van der Mark P, et al. MrBayes 3.2: efficient Bayesian phylogenetic inference and model choiceacross a large model space. Systematic biology. 2012;61(3):539–42.
  32. Lewis P O. A likelihood approach to estimating phylogeny from discrete morphological character data. Systematic biology, 2001, 50(6): 913-925.
